# A Novel Description of Immunodeficiency and Immune Dysregulation in a 14-Year-Old Girl with Noonan Syndrome 13

**DOI:** 10.1007/s10875-025-01881-3

**Published:** 2025-04-21

**Authors:** Saira Tabassum, Sarah Grün, Ben Molloy, Eppie Jones, Patrick G. Buckley, Rebecca Amet, Anthony M. McElligott, Derek G. Doherty, Stephan Ehl, Timothy Ronan Leahy

**Affiliations:** 1 Department of Paediatric Immunology, Children’s Health Ireland at Crumlin, Dublin, Ireland; 2 Faculty of Medicine, Center for Chronic Immunodeficiency, Medical Center-University of Freiburg, Freiburg, Germany; 3 Faculty of Biology, Albert-Ludwigs-University of Freiburg, Freiburg, Germany; 4 Genuity Science (Ireland), Cherrywood Business Park, Building 4, Dublin, Ireland; 5 John Durkan Leukaemia Laboratories, Trinity College, Dublin, Ireland; 6 Discipline of Immunology, Trinity Translational Medicine Institute, Trinity College, Dublin, Ireland; 7 Discipline of Paediatrics, School of Medicine, Trinity College, Dublin, Ireland

**Keywords:** Noonan syndrome 13, MAPK1, Ras-ERK, mTOR, Immunodeficiency

## To the Editor

We describe features of both immunodeficiency and immune dysregulation in a 14-year-old girl with Noonan Syndrome 13 due to an activating pathogenic variant in *MAPK1*.

## Case Report

### Clinical History

Our patient (P1) was delivered at term with no antenatal or post-natal complications. During infancy and early childhood, she was noted to have failure to thrive, neurodevelopmental delay, and facial features (hypertelorism, palpebral fissures, ptosis, low set posteriorly rotated ears, long philtrum) suggestive for Noonan Syndrome (NS). No pathogenic variant was identified upon sequencing of the *PTPN11* gene. She was diagnosed with growth hormone deficiency at the age of six years and commenced on replacement therapy to which she had a good clinical response with a height velocity of 6 cm/year at the age of 9 years. Her ultimate height was a little below the 0.4th centile for her age.

P1 was initially referred for clinical immunology evaluation at the age of seven years with a history of recurrent respiratory tract infections, recurrent herpes labialis, and chronic seborrheic dermatitis. Detailed immunological evaluation demonstrated panhypogammaglobulinemia and an impaired antibody response to pneumococcal vaccination (Table 1). She had no evidence of gastrointestinal lymphangiectasia on endoscopy, contrast studies, or ultrasound. She had no evidence of pulmonary lymphangiectasia on CT thorax. Lymphocyte immunophenotyping demonstrated subtle but persistent abnormalities (increased transitional B cells, reduced class-switched memory B cells) in the B cell compartment (Table 1). P1 was commenced initially on antibiotic prophylaxis and subsequently on immunoglobulin replacement therapy as well as prophylactic valaciclovir and topical anti-fungal treatment with good effect.

**Table 1:** Immunological laboratory characteristics

	Result (reference range)
**Lymphocyte immunophenotyping (values in cells/uL)**	
Total lymphocytes	1613 (1500–5800)
**T cells (CD3+)**	1253 (1200–2600)
*CD4 + T cells*	790 (650–1500)
*CD4 + CD45Ra + CCR7+ (naïve)*	289 (200–1700)
*CD4 + CD45Ra- CCR7+ (central memory)*	147 (120–740)
*CD4 + CD45Ra- CCR7- (effector memory)*	295 (5-210)
*CD4 + CD45Ra + CCR7-(terminally differentiated)*	58 (0–51)
*CD4 + HLA-DR+ (activated)*	1.3% (0.3-2)
*CD8 + T cells*	411 (370–1100)
*CD8 + CD45Ra + CCR7+ (naïve)*	254 (78–640)
*CD8 + CD45Ra- CCR7+ (central memory)*	18 (2–86)
*CD8 + CD45Ra- CCR7- (effector memory)*	101 (16–810)
*CD8 + CD45Ra + CCR7-(terminally differentiated)*	39 (35–420)
*CD8 + HLA-DR+ (activated)*	5.5% (0.9–3.6)
*TCR γδ + T cells*	6.9%
*TCR αß+ CD4-CD8- (double negative) T cells*	1.1%
**B cells (CD19+)**	524 (270–860)
*CD27-IgM + IgD+ (naïve)*	464 (133–389)
*CD27 + IgM + IgD+ (memory)*	49 (22–43)
*CD27 + IgM-IgD- (class switched memory)*	5 (16–31)
*CD21-low B cells*	8 (2–12)
*Transitional B cells*	45 (10–24)
**NK cells (CD16 + 56+)**	90 (100–480)
**Serum immunoglobulin levels (before therapy)**	
IgG (g/L)	0.85 (5.4–16.1)
IgA (g/L)	< 0.05 (0.5–2.4)
IgM (g/L)	0.33 (0.5–1.8)
**Vaccine specific antibody titres**	
Pneumococcus (# serotypes with protective titre > 0.35 ug/mL] post-vaccination)	1/12

Reference ranges as per Schatorje EJH et al., Scand J Immun 2011

P1 also had a history of chronic diarrhoea. At three years of age, a duodenal biopsy demonstrated an enteropathy consistent with coeliac disease. Her symptoms initially improved upon commencing a gluten-free diet, but she continued to have episodic symptoms of diarrhoea. Repeat endoscopy was undertaken when P1 was eight years of age because of persistent diarrhoea despite adherence to the gluten-free diet. She had evidence of a florid lymphocytic gastritis with lymphoid infiltration of gastric pits and surface epithelium (Supplementary Fig. 1). In the setting of her immunodeficiency phenotype, an alternative differential of autoimmune enteropathy rather than coeliac disease was considered. Gluten-containing foods were re-introduced into her diet and a trial of sirolimus therapy was instituted. Repeat endoscopy and biopsy undertaken after the trial of sirolimus (8 months with target trough level of 5–10 ng/ml) demonstrated an improvement in the appearance of the gastric mucosa, but subtle, re-emergent changes in the small bowel (Supplementary Fig. 2). P1 was, however, unable to tolerate adverse effects related to sirolimus. She ultimately had a good clinical response to re-institution of a gluten-free diet and oral sulphasalazine and remains on this treatment. P1 also developed a mild psoriatiform rash on the skin of her eyelids which responded well to low dose topical corticosteroid ointment.

**Fig. 1:**
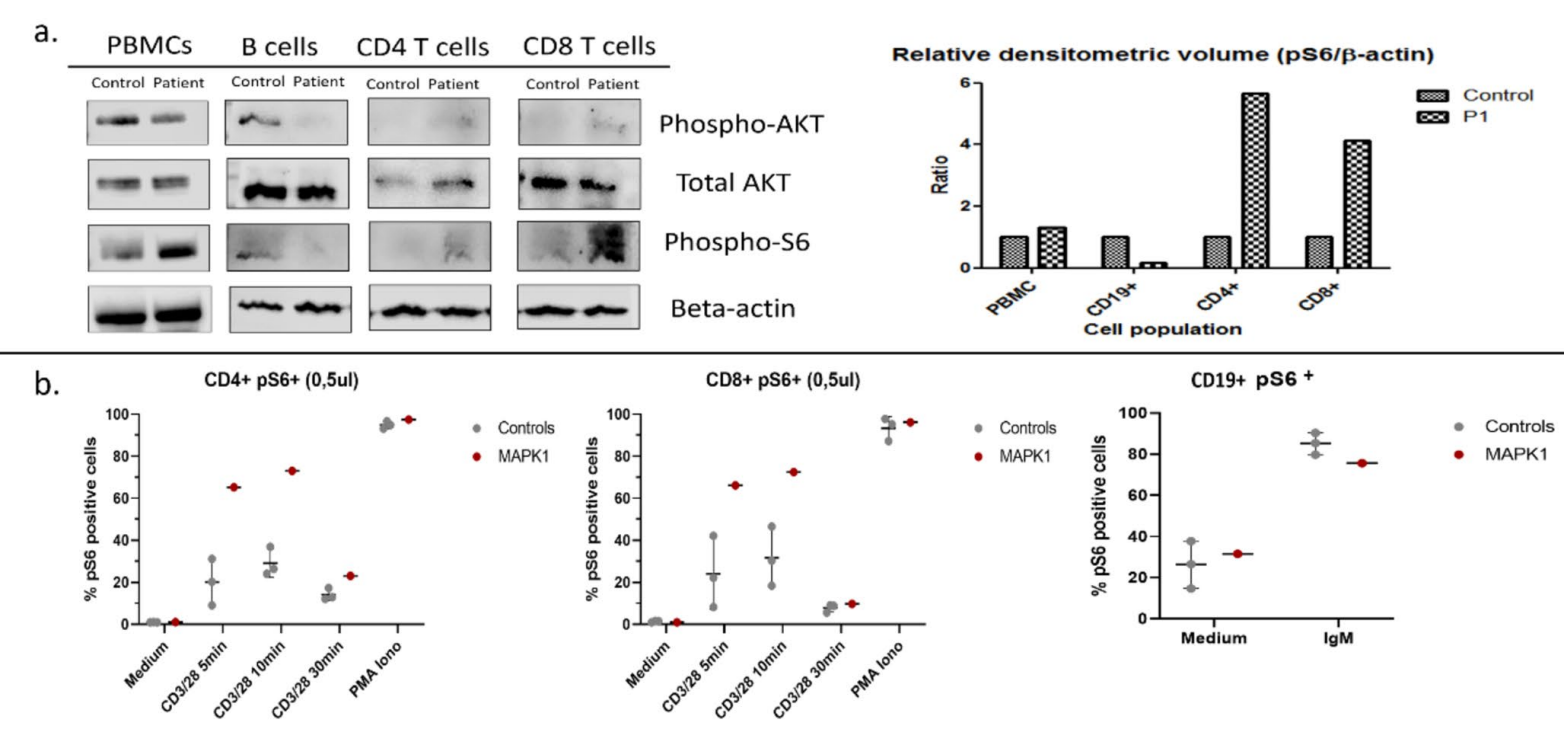
**a** demonstrates results of Western Blot comparing expression of pAKT, AKT, pS6 and β-actin in PBMCs, MACS-purified B-cells and T-cells from P1 and a healthy control (left panel) along with densitometry results (right panel). **b** shows a comparison of the relative proportion of cells expressing pS6 between P1 and healthy controls (x3) in CD4 + T-cells, CD8 + T cells and B-cells incubated in medium or with the indicated stimuli

Given the complex phenotype suggestive of a syndromic inborn error of immunity, whole genome sequencing (see supplementary data) was undertaken when P1 was nine years old. P1 was found to be heterozygous for a *de novo* pathogenic variant (c.238 C > T; p.His80Tyr) in *MAPK1 (aka ERK2)*, encoding mitogen activating protein kinase 1. A recent report by Motta *et al.* described activating pathogenic variants in *MAPK1* as causative for a neurodevelopmental disorder within the RASopathy clinical spectrum [1]. Mutations in *MAPK1* have subsequently been curated on OMIM (MIM:619087) as causative for Noonan Syndrome 13. The case series by Motta *et al.* included seven patients, among them a six-year-old boy with the identical variant in *MAPK1* as was identified in P1. In addition to the typical features seen in the Noonan Syndrome phenotype, two of the children in the case series had recurrent infection and one had features of inflammatory bowel disease. Functional studies undertaken by Motta *et al.* further demonstrated that increased MAPK1 phosphorylation associated with overexpression of the mutant protein led to enhanced phosphorylation of ribosome S6 kinases 1–3, established cytoplasmic substrates of the MAPK1 kinase. These experiments were undertaken in cell lines ectopically expressing individual *MAPK1* mutant alleles and primary fibroblasts from one of the identified patients. To investigate the impact of the *MAPK1* mutation on P1’s immune system, we compared pAKT and pS6 levels in PBMCs isolated from whole blood collected from P1 in comparison to healthy controls using Western Blot and flow cytometry (see supplementary data). We demonstrated increased pS6 expression in P1’s PBMCs and T-cells but not B cells by Western Blot (Fig. 1a); a similar pattern was observed in lymphocytes by flow cytometry (Fig. 1b; both by mean fluorescence intensity and by percentage positive pS6 + cells). A more extensive illustration of results is provided in the supplementary data.

## Discussion

Patients with pathogenic variants in the Ras-ERK pathway (Ras-opathies) can present with a broad array of clinical features, including features associated with inborn errors of immunity (IEI), predisposition to malignancy (JMML) and autoimmunity [2, 3]. An increased susceptibility to infection and/or hypogammaglobulinemia, however, has not been described before among NS patients aside from secondary hypogammaglobulinemia in patients with NS-related lymphangiectasia [4].

Mutations in genes (*PTEN*, *PI3KCD*, *PI3KR1*) associated with the PI3K/AKT/mTOR kinase pathway that lead to increased AKT phosphorylation and subsequently increased S6 kinase activity produce a clinical phenotype (activated PI3-kinase delta syndrome– APDS) that includes increased susceptibility to viral infection, lymphoma, hypogammaglobulinemia, and lymphoproliferation [5–8]. Increased pAKT has been demonstrated in activated T cells of patients with APDS [5–7]. Increased pAKT has also been demonstrated in unstimulated B cells from patients with APDS, allowing discrimination from patients with antibody deficiency of alternative aetiology [9]. Other IEI’s known to dysregulate the mTOR-S6 kinase pathway include LRBA deficiency and IEI’s related to mutations in genes associated with the CARD11-BCL10-MALT1 (CBM) signalosome [8]. These conditions together are sometimes referred to as “immune-TOR-opathies”.

**Fig. 2:**
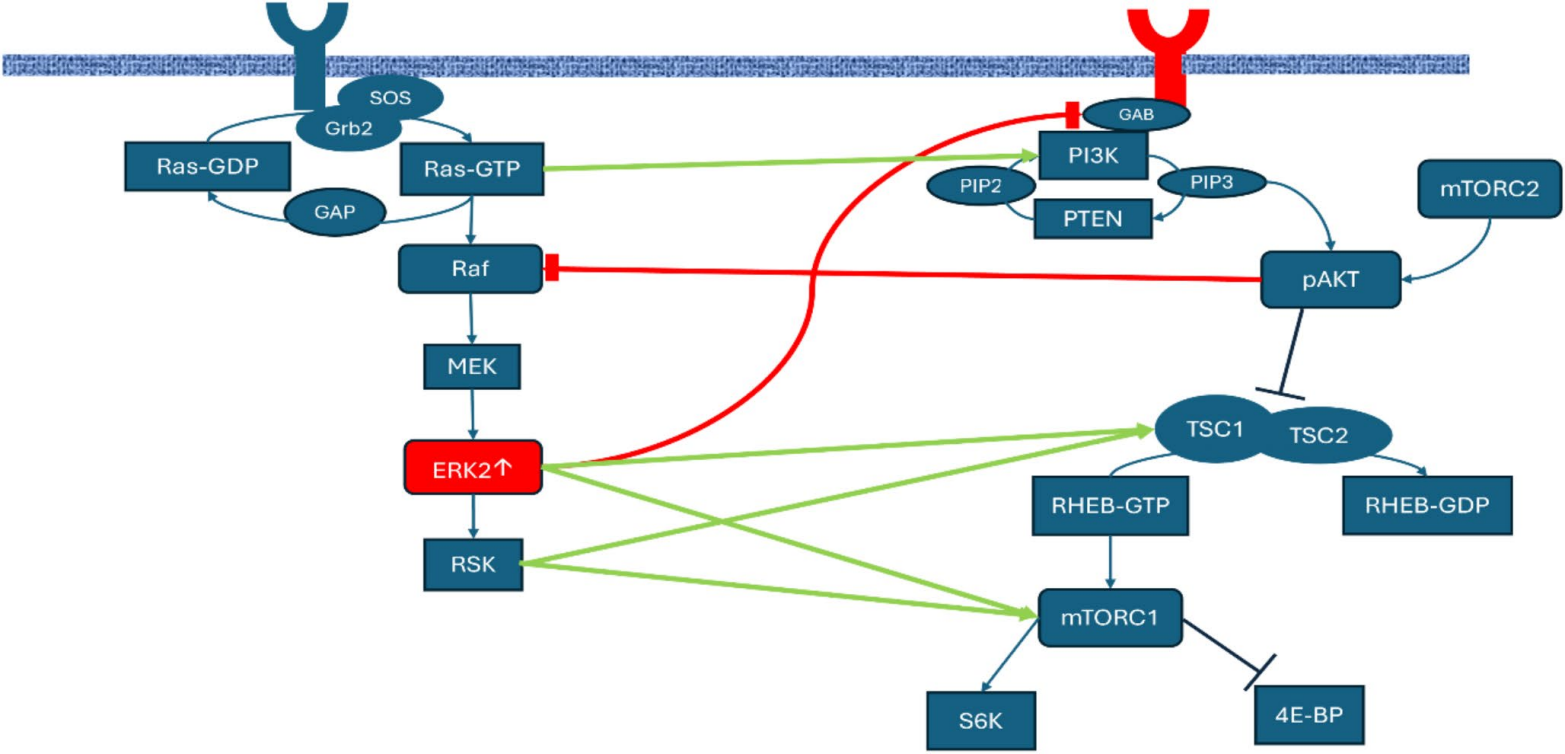
Pathway interaction. This figure. (adapted from Mendoza et al.[10]) illustrates how the Ras-ERK and PI3K-AKT-mTOR signalling pathways influence each other by cross inhibition (red lines) and cross-activation (green lines). Positive regulation is denoted as an arrow; negative regulation as a blunt-ended line. The MAPK1 gene encodes ERK2 which positively regulates the PI3K-AKT-mTOR pathway but also acts to negatively regulate the pathway upstream by phosphorylation of GAB

The Ras-extracellular signal-regulated kinase (Ras-ERK) and phosphatidylinositol 3-kinase- protein kinase B-mammalian target of rapamycin (PI3K-AKT-mTOR) signalling pathways are central to the control of cell survival, proliferation, metabolism, and response to extracellular cues [10]. There is evidence of intersection and cross regulation between these pathways (Fig. 2). The Ras-ERK pathway cross-activates PI3K-mTORC1 signalling by regulating PI3K, TSC2, and mTORC1, and increased activation of the RAS-ERK pathway can also lead to mTORC1 activity through ERK and ribosome S6 kinase signalling via the TSC complex [10]. Pathway convergence is also evident through the action of both pathways on S6 kinases, leading to increased cell survival and proliferation.

P1 had a combination of clinical and immunological features reminiscent of both Ras-opathy and immune-TOR-opathy patients. We hypothesise that the mutation in *MAPK1* leads to increased pS6 in T-cells, possibly due to an effect on both the Ras-ERK and PI3K-ATK-mTOR pathway caused by convergence between the two signalling pathways. In an analogy of the phenotype of patients with other immune-TOR-opathies, this may explain P1’s increased susceptibility to infection, hypogammaglobulinemia and immune dysregulation. While P1’s most prominent finding on initial clinical laboratory testing was hypogammaglobulinemia, the relative reduction in the proportion of class-switched memory B cells, the relative increase in transitional-B cells, and the relative sparing of serum IgM levels suggest a defect in T-cell help rather than an intrinsic B-cell compartment disorder. Evaluation of the immunological phenotype in other patients with Noonan Syndrome 13 is needed to clarify this more definitively.

To conclude, we suggest sequencing of *MAPK1* in patients presenting with Noonan Syndrome phenotype and features of immunodeficiency/immunodysregulation, and careful evaluation of the immune system in patients with activating mutations in *MAPK1* and other genes on the Ras-ERK pathway.

## Electronic Supplementary Material

Below is the link to the electronic supplementary material.

Supplementary Material 1

## Data Availability

No datasets were generated or analysed during the current study.
