# Supplementary material for: A Novel Description of Immunodeficiency and Immune Dysregulation in a 14-Year-Old Girl with Noonan Syndrome 13
Source: J Clin Immunol. 2025 Apr 21;45(1):87. doi: 10.1007/s10875-025-01881-3 (PMC12011885; doi:10.1007/s10875-025-01881-3)
Supplement: Supplementary file 1 — Supplementary Material 1 [file 10875_2025_1881_MOESM1_ESM.docx]

**Immunodeficiency and immune dysregulation in a 14-year-old girl with Noonan Syndrome 13**

Saira Tabassum MD^1^, Sarah Grun ^2,3^, Ben Molloy PhD^4^, Eppie Jones PhD^4^, Patrick G. Buckley FRCPath PhD^4^, Rebecca Amet PhD^5^, Anthony M. McElligott PhD^5^, Derek G Doherty PhD^6^, Stephan Ehl^2^, Timothy Ronan Leahy MD PhD^1,7^

^1^Department of Paediatric Immunology and ID^1^, Children’s Health Ireland at Crumlin, Dublin, Ireland

^2^ Faculty of Medicine, Center for Chronic Immunodeficiency, Medical Center-University of Freiburg, Freiburg, Germany.

^3^ Faculty of Biology, Albert-Ludwigs-University of Freiburg, Freiburg, Germany.

^4^Genuity Science (Ireland), Cherrywood Business Park, Building 4, Dublin, Ireland

^5^ John Durkan Leukaemia Laboratories, ^6^Discipline of Immunology, Trinity Translational Medicine Institute, and ^7^Discipline of Paediatrics, School of Medicine, Trinity College Dublin, Dublin, Ireland

**Supplementary Materials**

**Supplementary methods**

**DNA Isolation**

Genomic DNA was isolated from whole peripheral blood using Qiagen’s Flexigene precipitation chemistrry on FlexSTAR PLUS totally automated isolation instrument (AutoGen Inc, Holliston, MA, USA), according to the manufacturer’s protocol. Sample purity (using UV absorbance) and quantity (using PicoGreen® dsDNA quantitation assay) were measured using the Varioskan™ LUX multimode microplate reader (Thermo Fisher Scientific Inc., Waltham, MA, USA). Sample size and integrity were assessed using the 4200 TapeStation System (Agilent, Santa Clara, CA, USA). All liquid handling during sample preparation were automated using a Microlab STAR Liquid Handling System (Hamilton Robotics, Reno, NV).

**Whole Genome Sequencing & Analysis**

Whole genome sequencing was performed using Illumina TruSEQ DNA PCR-free whole-genome sequencing with 2x150bp pair-end sequencing reads (Illumina Inc., San Diego, CA, USA), following the manufacturer’s protocol. The generated library was sequenced using the NovaSeq-6000 instrument (Illumina Inc., San Diego, CA, USA). The minimum mean sequencing coverage was 30-fold, with a minimum of 95% of the target bases covered at least 10 times. FASTQ data was aligned using the Burrows-Wheeler aligner (BWA) to the GRCh38/hg38 reference genome build, and variants were called using the Genome Analysis Toolkit (GATK). These variants, annotated for functional effect by the Variant Effect Prediction (VEP-Ensembl, Version 96.2) were analysed on the WuXi NextCode Clinical Sequence Analyser (CSA). Alamut Visual version 2.12 (Interactive Biosoftware) was used to analyze the impact of the candidate variants, which showed alignment of orthologues genes and includes several protein-function prediction tools such as SIFT, PolyPhen-2 and Mutation Taster.

**Sanger Sequencing**

Sanger sequencing was performed using the BigDye® Direct Cycle Sequencing Kit in combination with the BigDye XTerminator™ Purification Kit (Applied Biosystems, Foster City, CA, EUA) and the Applied Biosystems SeqStudio™ Genetic Analyzer. Sequencing data were analyzed using the software GeneStudioTM Professional Edition Version 2.2.0.0 (GeneStudio, Inc) and FinchTV Version 1.4 (Geospiza, Inc).

**Western Blot**

Immunoblotting was undertaken in the John Durkan Leukemia Laboratory, TCD, Dublin on Peripheral blood mononuclear cells (PBMCs) and both T cell and B cell compartments on blood collected from P1 and from two healthy controls in CHI at Crumlin. The experiments were undertaken in duplicate. PBMCs were prepared from whole blood by standard density gradient centrifugation over Lymphoprep^TM^ (Axis-Shield). Cells were stained with fluorochrome-labelled monoclonal antibodies specific for CD3, CD4, CD8 and CD19 (BioLegend) and the CD3^-^ CD19^+^ cells (B cells), CD3^+^ CD4^+^ cells (CD4 T cells) and CD3^+^ CD8^+^ (CD8 T cells) were isolated using a BD FACSMelody™ Cell Sorter (BD Biosciences). Patient and control cell pellets were re-suspended in cold cell lysis buffer (radio-immunoprecipitation assay buffer, Sigma) supplemented with 1% phosphatase inhibitor cocktail 2 and 3 (Sigma) and 10% protease inhibitor (Roche).

Cells were lysed for 30 minutes on ice. Protein concentration was then determined by BCA assay. Lysates were boiled with Laemmli sample buffer (Sigma) for 10 min at 90°C. Equal volumes of cell lysates were resolved by sodium dodecyl sulphate polyacrylamide gel electrophoresis (SDS-PAGE) and transferred onto PVDF transfer membrane (Sigma). Membranes were blocked with 5% non-fat milk and probed with primary antibodies for phospho-AKT S473 (Cell Signalling) and phospho-S6 ribosomal protein S240/244 (Cell Signaling). The membrane was then stripped with Restore™ western blot stripping buffer (Sigma) and blocked again with 5% milk. The membranes were then probed with antibodies specific for total AKT (Cell Signalling) and beta-actin (Sigma) to confirm equal loading.

**Flow Cytometry**

PBMCs from P1 and from two travel controls were sent from CHI @ Crumlin, Dublin, Ireland to Professor Ehl’s laboratory in CCI Freiburg for flow cytometry. An “in-house” control was also used. For assessment of phosphorylation of S6 in T cells, PBMCs were rested overnight in 5% CO_2_ at 37°C. Cells were stained for 20 minutes at 4°C with NearIR–fixable Live/Dead (Invitrogen, #L10119). For stimulation, cells were incubated for 15 minutes with 2.5 µg/ml anti-CD3 (BioLegend, #317320) and 2.5 µg/ml anti-CD28 (eBioscience) antibodies on ice. NeutrAvidin (Thermo Scientific, #31000) was added to a final concentration of 50 µg/ml and cells were incubated for 15 minutes on ice.

The samples were then transferred to 37°C and incubated for 5 minutes, 10 minutes, or 30 minutes respectively. As a positive control, cells were stimulated with PMA/Ionomycin (0.05µg/ml, Sigma Aldrich, #P1585; 1µg/ml, Sigma Aldrich, #I0634) for 10 minutes at 37°C. Cells were fixed using BD Cytofix Fixation buffer (BD Biosciences, #554655; p-S6) for 10 minutes at 37°C. Permeabilisation was done using BD Phosflow Perm Buffer III (BD Biosciences, #558050) for 30 minutes on ice. Intracellular and extracellular staining was done for 30 minutes at room temperature . Data was acquired on a Navios (Beckman Coulter). Phosphorylation of S6 in B cells was performed as previously described by Harder *et al.*[1] with the following adaptations: thawed PBMCs were rested for 4h at 37°C. Fixation/Permeabilisation and staining was performed as described above. Data was acquired on a LSR Fortessa (BD Biosciences).

Antibodies used for FACS stainings in this study: CD3 PerCP (BD Pharmingen, #45766), CD4 PC7 (Beckman Coulter, #737660), CD8 V450 (BD Horizon, #560347), CD19 BV421 (BioLegend, #302234), CD21 PC7 (BioLegend, #354912), CD27 BV605 (BD Horizon, #562655), CD38 PerCP Cy5.5 (BioLegend, #303522), Phospho-Akt (Ser473) Alexa Fluor 488 (cell signalling, #4071S), Akt (pS473) PE (BD Phosflow, #561671), IgM APC-Cy7 (BioLegend, #314520), S6 (pS240) Alexa Fluor 647 (BD Phosflow, #560432).

**Supplementary Figures**

**
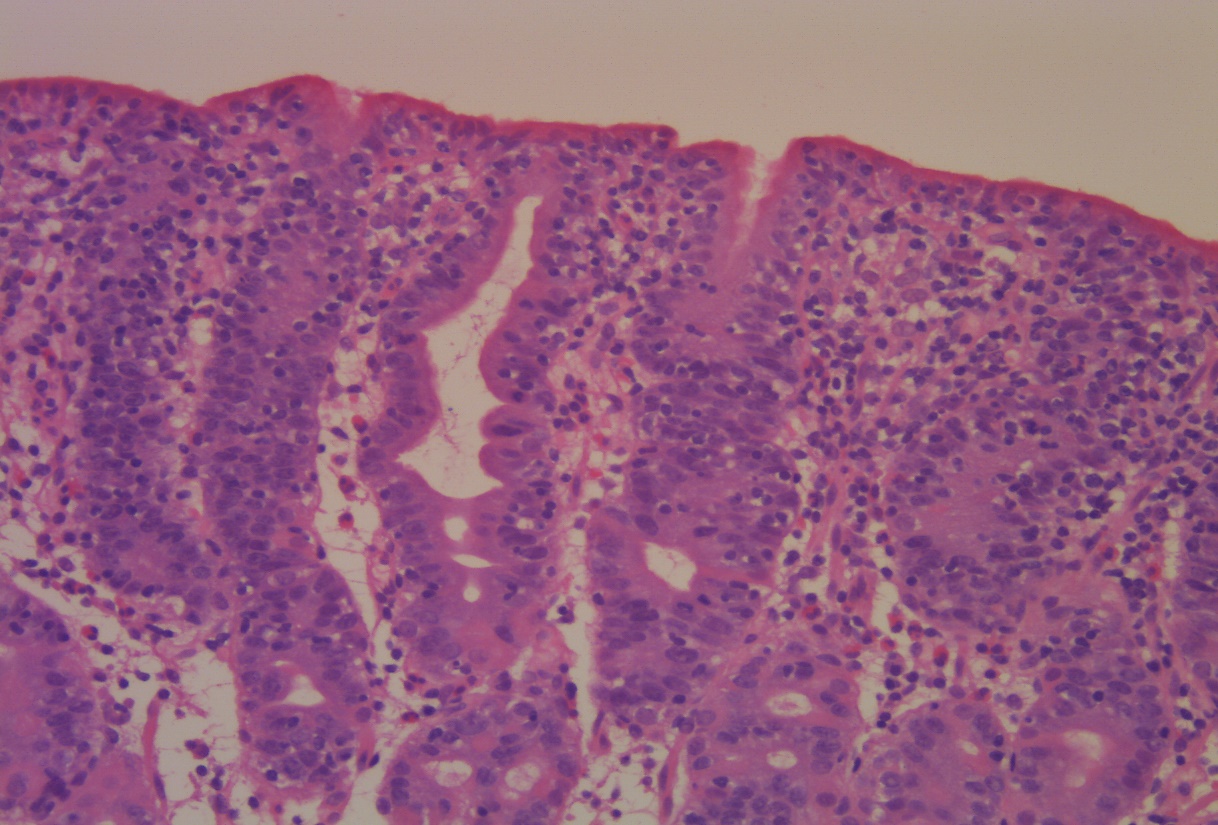
**

**Supplementary Figure 1:** Gastric mucosal biopsy showing florid lymphocytic gastritis with lymphoid infiltrate of gastric pits and surface epithelium


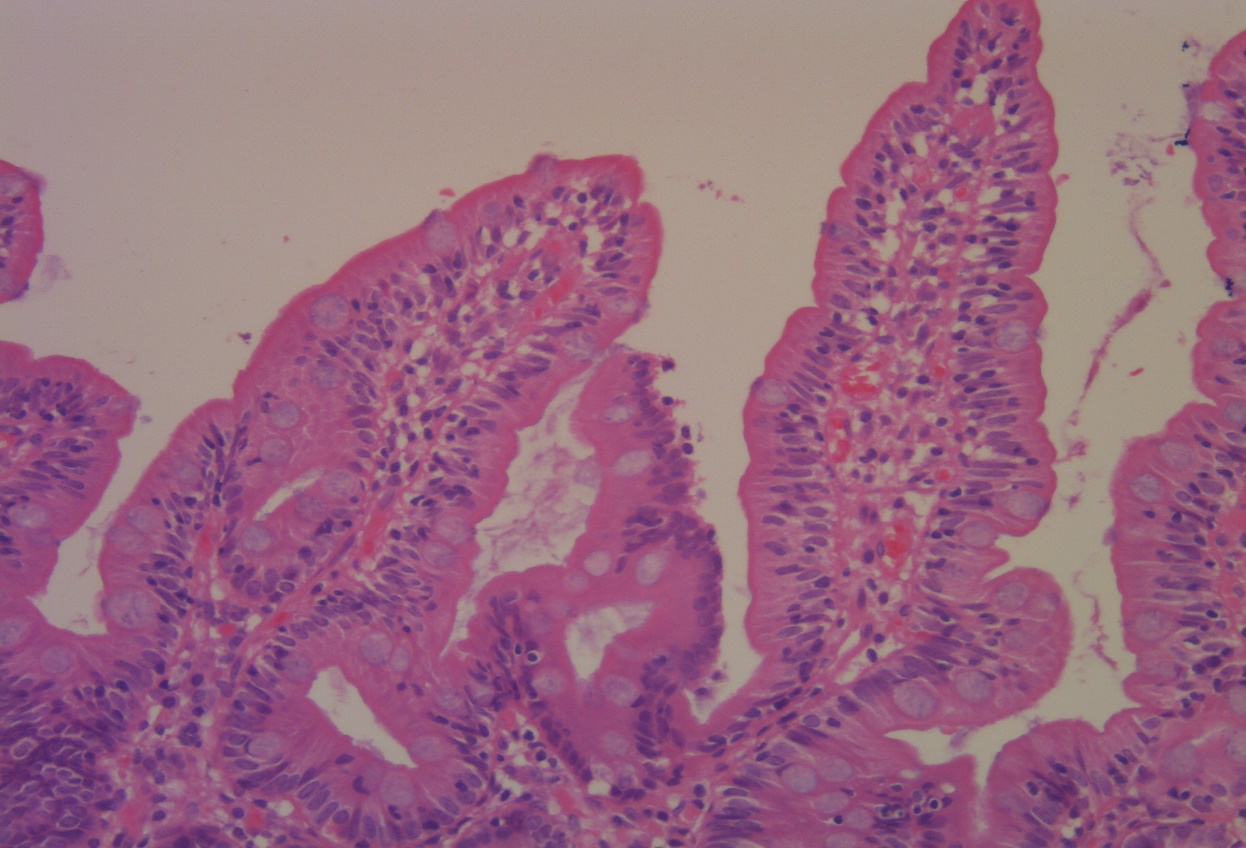


**Supplementary Figure 2:** Small bowel biopsy showing subtle enteropathy with preserved villous architecture but increased number of intra-epithelial lymphocytes in surface epithelium.

**Supplementary results**

**Flow cytometry - Gating strategy**

The gating strategy for T-cells was as follows.


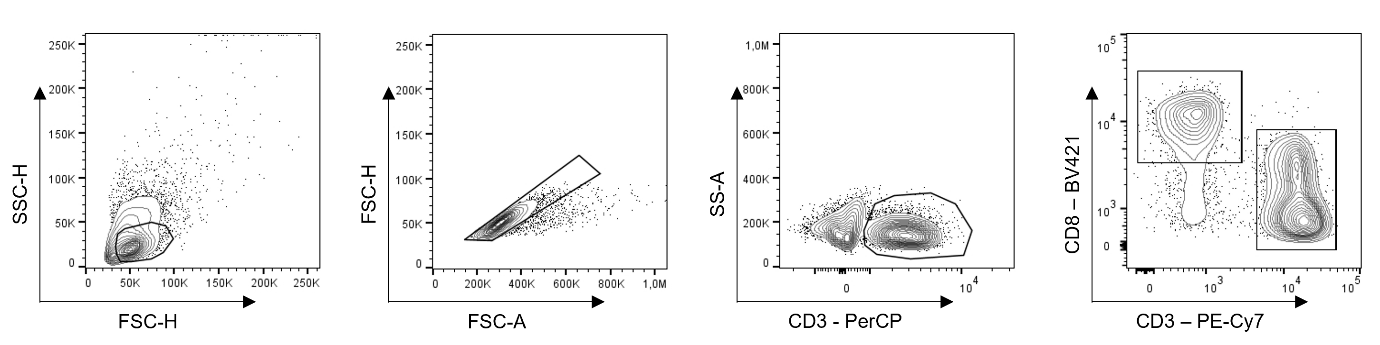


The gating strategy for B-cells was as follows.


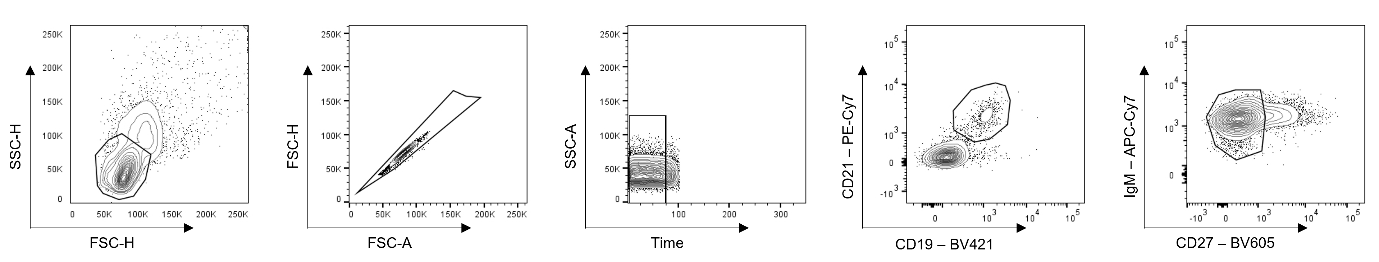


Comparison of MFI of pS6+ cells from P1 and healthy controls as follows.


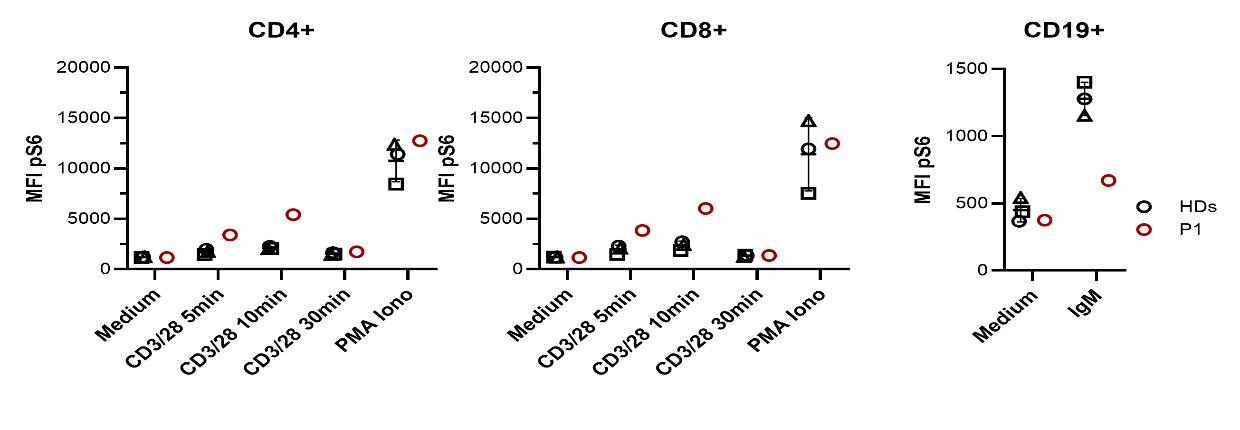


Comparison of pAKT in CD4+ T cells between P1 and healthy controls as follows.


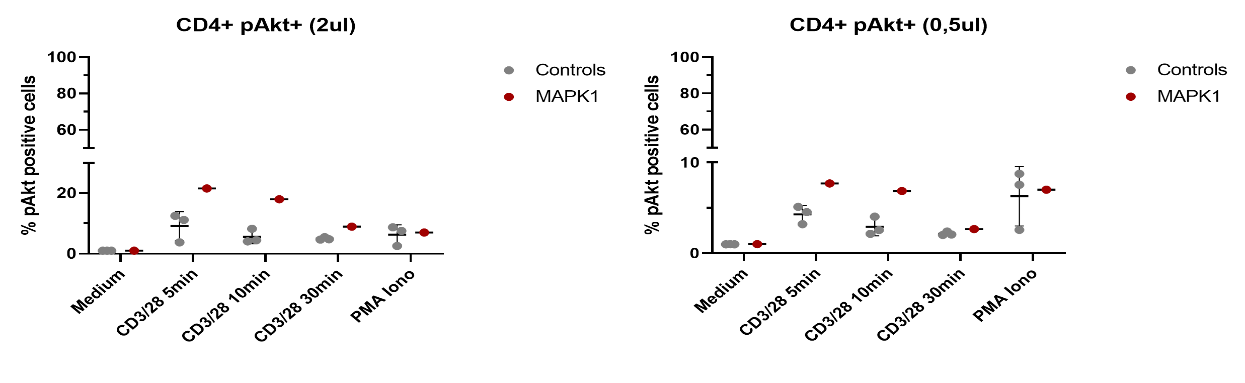


Comparison of pAKT and pmTOR in B cells of P1 and healthy controls as follows.


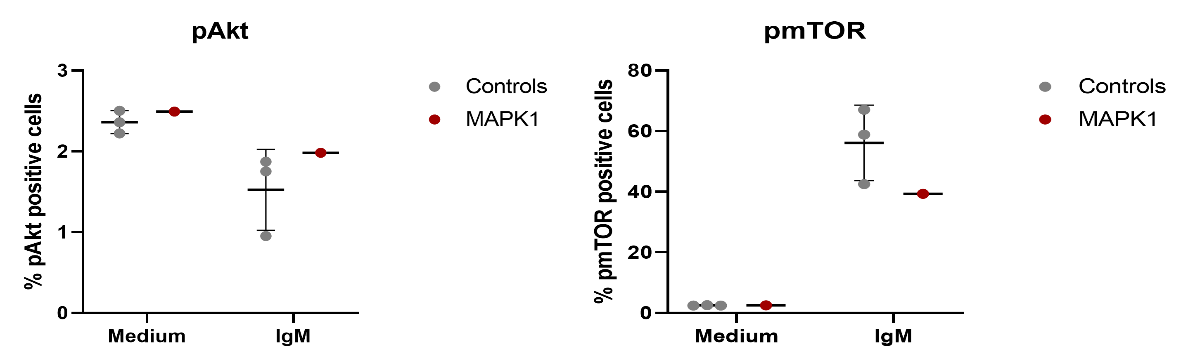


**References**

1. Harder, I., et al., *Dysregulated PI3K Signaling in B Cells of CVID Patients.* Cells, 2022. **11**(3).
